# Optimizing somatic embryogenesis in Indonesian cassava genotypes: Effects of 2,4-D or Picloram supplementation and explant type

**DOI:** 10.5511/plantbiotechnology.25.1003b

**Published:** 2026-03-25

**Authors:** Febriana Dwi Wahyuni, Ahmad Fathoni, Dewi Sukma, Sintho Wahyuning Ardie, Ima Mulyama Zainuddin, Baoxiu Qi, Sudarsono Sudarsono

**Affiliations:** 1 Biotechnology Department, Faculty of Health Sciences, Esa Unggul University, Jl. Arjuna Utara No. 9, West Jakarta 11510; 2 Plant Breeding and Biotechnology Program, Department of Agronomy and Horticulture, Faculty of Agriculture, IPB University, Jln. Meranti, IPB Dramaga Campus, Bogor 16680, West Java, Indonesia; 3 Research Center for Applied Microbiology, National Research and Innovation Agency (BRIN), Bogor 16911, West Java, Indonesia; 4 Research Center for Genetic Engineering, National Research and Innovation Agency (BRIN), Bogor 16911, West Java, Indonesia; 5 School of Pharmacy and Biomolecular Sciences, Faculty of Science, Liverpool John Moores University, Liverpool L3 3AF, England, UK

**Keywords:** auxin, callus, *Manihot esculenta*, somatic embryo, somatic embryogenic callus

## Abstract

Modern biotechnological approaches in technology for cassava propagation depend on somatic embryogenic calli (SEC) induction and somatic embryo (SE) regeneration techniques. Consequently, it is essential to develop SEC induction and SE regeneration for cassava genotypes. By assessing the effects of different auxins and explant types, this study aims to develop efficient techniques for inducing cassava SEC and SE. Callus induction medium (CIM) supplemented with 10 mg l^−1^ 2,4-dichlorophenoxyacetic acid (2,4-D) or 12 mg l^−1^ Picloram was used to cultivate five different types of cassava explants. Subsequently, the induced calli were maintained on CIM until the formation of SEC, which were sub-cultured on the same CIM to promote proliferation and induce SE formation. These SEs subsequently germinated and developed into shoots, which were rooted to produced complete plantlets . These findings indicate that culturing-induced axillary bud explants on CIM supplemented with 12 mg l^−1^ Picloram is an effective method for inducing SEC in cassava. Histological analyses and cryogenic electron microscopy observations confirmed the development of SECs and SEs on CIM. Our finding, in which up to 202.3 SEs were regenerated from 10 induced axillary bud explants on CIM supplemented with 12 mg l^−1^ Picloram in the Menti genotype, highlights the effects of auxin and explant types on SEC and SE induction. Although further research is required, the methods developed in this study will contribute to the successful breeding and micropropagation of cassava.

## Introduction

Cassava is a staple food for more than 800 million people in tropical and subtropical regions, particularly Africa, Asia, and Latin America (Burns et al. 2010). It is a dependable crop that can tolerate poor soil and drought conditions. Cassava starchy roots provide an important carbohydrate source that contributes substantially to daily nutritional requirements (Buttibwa et al. 2022). In Indonesia, cassava is widely consumed, and there is a growing demand for this crop plant as a raw material for industrial use (Yelli et al. 2023). The increased cassava demand highlights the future need for a larger supply of cassava planting materials, which the development of tissue culture technologies could address.

Despite its importance, the cultivation of cassava faces multiple challenges, including limited genetic diversity, vulnerability to pests and diseases, and the scarcity of suitable breeding materials (Ntui et al. 2024). The extended growth cycle, low reproductive success, highly heterozygous genetic composition, and restricted genetic base complicate conventional cassava breeding approaches (Slameto et al. 2023). Consequently, developing biotechnological strategies for enhancing the genetic diversity of cassava is considered essential for addressing these constraints (Chavarriaga-Aguirre et al. 2016).

Among these approaches, somatic embryogenesis is an important initial step in cassava biotechnology. Somatic embryogenesis provides numerous essential benefits, including the rapid and economic propagation of planting materials, the production of disease-free plants, and genetic transformation (Nkaa et al. 2013). Moreover, developing robust somatic embryogenesis and plantlet regeneration methods will facilitate using genome editing tools in cassava breeding (Nyaboga et al. 2015).

The induction of somatic embryos (SEs) in cassava is dependent on several factors, including the composition of the culture medium, types and concentrations of supplemented auxins in the medium, source tissues used as explants, and the genetic makeup of cassava plants (Fletcher et al. 2011; Syombua et al. 2019). Although Murashige and Skoog (MS) medium is widely utilized for the induction of cassava SEs (Bull et al. 2009; Danso et al. 2010), supplementing the medium with PGRs is the most important step in attaining consistent SE production and subsequent plantlet regeneration (Danso et al. 2010; Syombua et al. 2019).

Auxins are required to initiate this process, whereas cytokinins stimulate SE development and plantlet regeneration (Bull et al. 2009; Syombua et al. 2019). Studies have revealed that 2,4-dichlorophenoxyacetic acid (2,4-D), Picloram, and naphthalene acetic acid (NAA) can induce SE in cassava (Berhanu and Feyissa 2020; Syombua et al. 2019), with 2,4-D and Picloram being established to outperform NAA in this regard (Mongomake et al. 2015).

The successful induction of SEs has been achieved in numerous African cassava genotypes using media containing 2,4-D or Picloram (Magambo et al. 2024; Mongomake et al. 2015). Similarly, Picloram has been established to contribute to the induction of SEs and the subsequent development of friable embryogenic calli (FEC) in Asian cassava genotypes, such as KU50 and SC8 (Utsumi et al. 2022; Wang et al. 2022). These latter calli are particularly desirable because of their loose, crumbly texture and capacity to grow into entire plants. Their regenerative capacity and ease of use make FEC invaluable tools for plant transformation and regeneration research (Bull et al. 2009). Previous studies in this regard have revealed the effective induction of SEs in the Indonesian cassava genotype UJ-3 and BW-1 using MS medium supplemented with Picloram and NAA (Yelli et al. 2023), and further research has demonstrated that Picloram effectively induces SE in some Indonesian cassava genotypes, including Kaspro and Gajah (Slameto et al. 2023). However, a key challenge is that the efficacy of cassava SE induction differs substantially depending on genotype (Magambo et al. 2024; Mongomake et al. 2015), thereby highlighting the need for the optimization of protocols for the development of new cassava genotypes that prioritize the use of 2,4-D or Picloram as auxins.

In addition to the composition of the culture medium, explant type can have a significant influence on the success of cassava somatic embryogenesis induction, among which axillary buds, leaf lobes, petioles, stems, and young leaf explants have previously been used (Bull et al. 2009; Fletcher et al. 2011; Yelli et al. 2023), with axillary buds, leaf lobes, and young leaves being the most commonly reported explants (Bull et al. 2009; Fletcher et al. 2011; Yelli et al. 2023). Given that cassava somatic embryogenesis is genotype dependent (Magambo et al. 2024; Mongomake et al. 2015), evaluating different explant types is essential for optimizing the production of SEs to develop new cassava varieties.

This study achieved somatic embryogenesis and plant regeneration through a sequential process that commenced with callus induction, progressed to the production of somatic embryogenic calli (SEC), advanced to the development of somatic embryos (SE), and culminated in the regeneration of entire plants. The three primary phases of this study include explant and auxin type selection, somatic embryo induction, and plantlet regeneration in three genotypes of Indonesian cassava. Selecting diverse cassava genotype allows the identification of cassava genotype with the best SE induction potential. The findings of this research will contribute to enhancing biotechnology-based cassava breeding strategies and enable large-scale plant propagation using the optimized tissue culture protocols.

## Materials and methods

### Plant materials

This study used three locally grown cassava genotypes (Manggu, Menti, and Nangka), received as in vitro collections from the Soekarno Integrated Science Area, BRIN, Cibinong, West Java, Indonesia. We used these collections as sources of explants to avoid sterilization during culture initiation. Manggu and Menti are white-fleshed genotypes. In contrast, Nangka has yellow-fleshed tuberous roots. Manggu is a high-yielding genotype that produces cassava chips and modified cassava flours (Sudarmonowati et al. 2018). Given its low cyanide content, Menti is used for fresh consumption (Sudarmonowati et al. 2018). Nangka, rich in carotenoids, is used in traditional Indonesian food (Sudarmonowati et al. 2020).

### Induction of somatic embryogenic callus and somatic embryo

The induction of somatic embryogenic callus (SEC) was performed using five types of explants, namely, induced axillary buds, leaf lobes, leaves, petioles, and stems. The leaf lobe, leaf, petiole, and stem explants were cut to a size of 5 mm and cultured on callus induction medium (CIM) containing MS basal medium (Murashige and Skoog 1962), supplemented with 2% sucrose, 0.2% Gelrite (Phytotech), and either Picloram (12 mg l^−1^) or 2,4-dichlorophenoxy acetic acid (2,4-D, 10 mg l^−1^). The leaf lobes explant were carefully excised from young leaves of 3–4-week-old in vitro cassava plantlets. Axillary bud explants were obtained by cutting internodes (5 mm) from 4-week-old cassava plants in vitro. The internodes were cultured horizontally with the axil upper-most on cassava axillary medium (CAM) comprising an MS basal medium, supplemented with 2 µM CuSO_4_, 2% sucrose, 0.2% gelrite, and 10 mg l^−1^ benzyl amino purine (BAP), and incubated in the dark for 3 to 4 days at 28°C for axillary bud activation, as previously described (Atehnkeng et al. 2006; Bull et al. 2009; Zainuddin et al. 2012). The enlarged axillary buds were removed using a sterile syringe needle under a binocular microscope. The explants were transferred to Petri dishes containing organized embryogenic structure (OES) induction medium with MS basal medium supplemented with 2 µM CuSO_4_, 2% sucrose, 0.2% Gelzan (Merck), and either Picloram (12 mg l^−1^) or 2,4-D (10 mg l^−1^). The Picloram and 2,4-D concentrations used in the present study were based on those previously reported (Bull et al. 2009; Fletcher et al. 2011; Mongomake et al. 2015). Having been maintained in the dark for 2 weeks at 28°C, all cultures were subsequently continually sub-cultured onto the same fresh medium at 2-weekly intervals and maintained under the same conditions to promote SEC propagation and secondary SE formation. The variables observed at the embryogenesis induction stage were the percentage of callus-forming explants, percentages of somatic embryo-forming calli, and the total number of somatic embryos. SEC is characterized by an irregular, clumped shape with a rough, granular surface. SE, on the other hand, is characterized by a globular shape, a smooth, dense surface, and a bright white or slightly yellowish color. Callus-forming explant refer to swelling or bulging at cut ends and the surface texture is rough, irregular, and compact. Callus formation was calculated by scoring the callus in two weeks of placing explants on CIM supplemented with Picloram or 2,4-D. Compact and nodular calli identified as SEC were selected for further culture. Somatic embryo-forming calli refer to compact, nodular structures that appeared approximately four weeks after the explants were cultured on the CIM containing Picloram or 2,4-D. In contrast to non-embryogenic calli, which tended to be watery, soft and lacked organized structures. The total number of SEs was determined by summing the number of SEs formed per explant, assessed six weeks after culture on CIM. After that, mature somatic embryos were placed in regeneration medium to promote germination and the growth of shoots and roots, resulting in whole plantlets.

### Regeneration of somatic embryo

For SE maturation, SE was placed on MS+NAA (MSN) medium comprising MS basal medium supplemented with 2% sucrose, 0.2% Gelzan, and 1 mg l^−1^ NAA. The SE maturation cultures were incubated for 7–10 days under a 16-h photoperiod at 28°C. Having developed into greenish tube-like structures, the mature SEs were maintained on MSN until they had produced mature cotyledons. In the case of cassava, mature SEs do not germinate readily in plantlets, with some of the total numbers of SEs germinating into green knobs designated cotyledonary-stage somatic embryos. The percentage of total number of SE developing into green knobs were calculated as the cotyledonary stage of somatic embryo formation (CSEF) using the following equation: CSEF (%)=(the number of cotiledonary-stage SE/the number of SE)×100%, assessed four weeks after culture on MSN.

To promote shoot elongation from shootlets, mature SEs with enlarged cotyledons were subsequently transferred to a cassava elongation medium (CEM) comprising MS basal medium supplemented with 2 µM CuSO_4_, 2% sucrose, 0.2% Gelrite, and 0.4 mg l^−1^ BAP. The cultures were maintained in Petri dishes until shoots (shoot-forming somatic embryo) had developed. Elongated shoots were cut and transferred to glass jars containing cassava basic medium (CBM) comprising MS basal medium, supplemented with 2 µM CuSO_4_, 2% sucrose, and 0.3% Gelrite, and maintained under a 16-h photoperiod at 28°C. The percentages of cotyledonary stage of SE formation developing into shoot-forming SE were calculated as the somatic embryos to shoot conversion (SESC), using the following equation: SESC (%)=(the number of shoot-forming SE/the number of cotyledonary SE formation)×100%, assessed eight weeks after culture on CEM. The plantlets were then transferred to MS medium for shoot elongation. Shoot height was measured four weeks after subculture on MS medium.

We prepared vermiculite medium in 24-cell trays and soil medium in polybags for plantlet acclimatization. To minimize root damage, plantlets were sub-cultured on CBM with a reduced content of Gelrite (2–2.5 g l^−1^). After 8–14 days, the plantlets were gently removed and placed in vermiculite trays covered with a transparent lid, in which they were maintained for 6 days. Thereafter, the lid was gradually opened to facilitate acclimatization to the ex vitro conditions, and having acclimatized, the plantlets were transferred to a growth chamber for 1–2 weeks and subsequently to a glasshouse (28–30°C, >50% humidity, 16-h daylight). After 1 week, the cassava plants were transferred to polybags (25×25 cm) containing potting mixes and watered daily for optimal growth.

### Histological analysis

Explants that developed different somatic embryonic structures were collected, and different stages of SE formation were selected for histological analysis. Samples were initially fixed in FAA [formaldehyde (5 ml, 37%), glacial acetic acid (5 ml), ethanol (90 ml, 70%)] for 24 h. Subsequent dehydration was carried out using a series of increasing ethanol concentrations (70, 80, 95, and 100%; 1 h for each concentration). The dehydrated tissues were thereafter processed using an alcohol-xylol series and embedded in paraffin (58°C). Sections (8 µm thick) were cut using a Leica RM2255 rotary microtome (Wetzlar, Germany), fixed on glass slides, and stained with hematoxylin and eosin (Liu et al. 2023). Histological observations of SEs at different stages of development were performed under a light microscope (Nikon, United States).

### Cryogenic electron microscopy

Two- and four-week-old SEC cultures were evaluated by cryogenic electron microscopy (cryo-EM). Imaging was conducted using a Dual-Beam FIB Aquilos2 scanning electron microscope (Thermo Fisher, Waltham, Massachusetts, USA), equipped with a field emission source operated at low temperatures (<0°C). The SEC samples were mounted on stubs using a sample medium and transferred under a high vacuum from the preparation station to the Aquilos2 chamber. The microscope stage facilitated a 360° rotation and tilting at different angles, providing optimal imaging flexibility. A thin platinum coating (∼5 nm) was applied to the samples for 15 s at a current of 30 mA using an integrated retractable sputter coater to enhance surface conductivity. Imaging was performed using an electron beam at a current of 25 pA and voltage of 2 kV, maintaining a working distance of 9–15 mm to ensure high-resolution observations without removing the samples during the process.

### Statistical analysis

The experiment was based on a completely randomized design with three replicates. Each experimental unit consisted of 10 explants. Therefore, each treatment combination consisted of 30 explants. Statistical analysis was conducted using an analysis of variance (ANOVA), and Tukeys honest significance difference test (at the α=5% level) was used to compare means.

## Results

### The effect of genotype, explant and auxin types on SE induction

**Figure 1:**
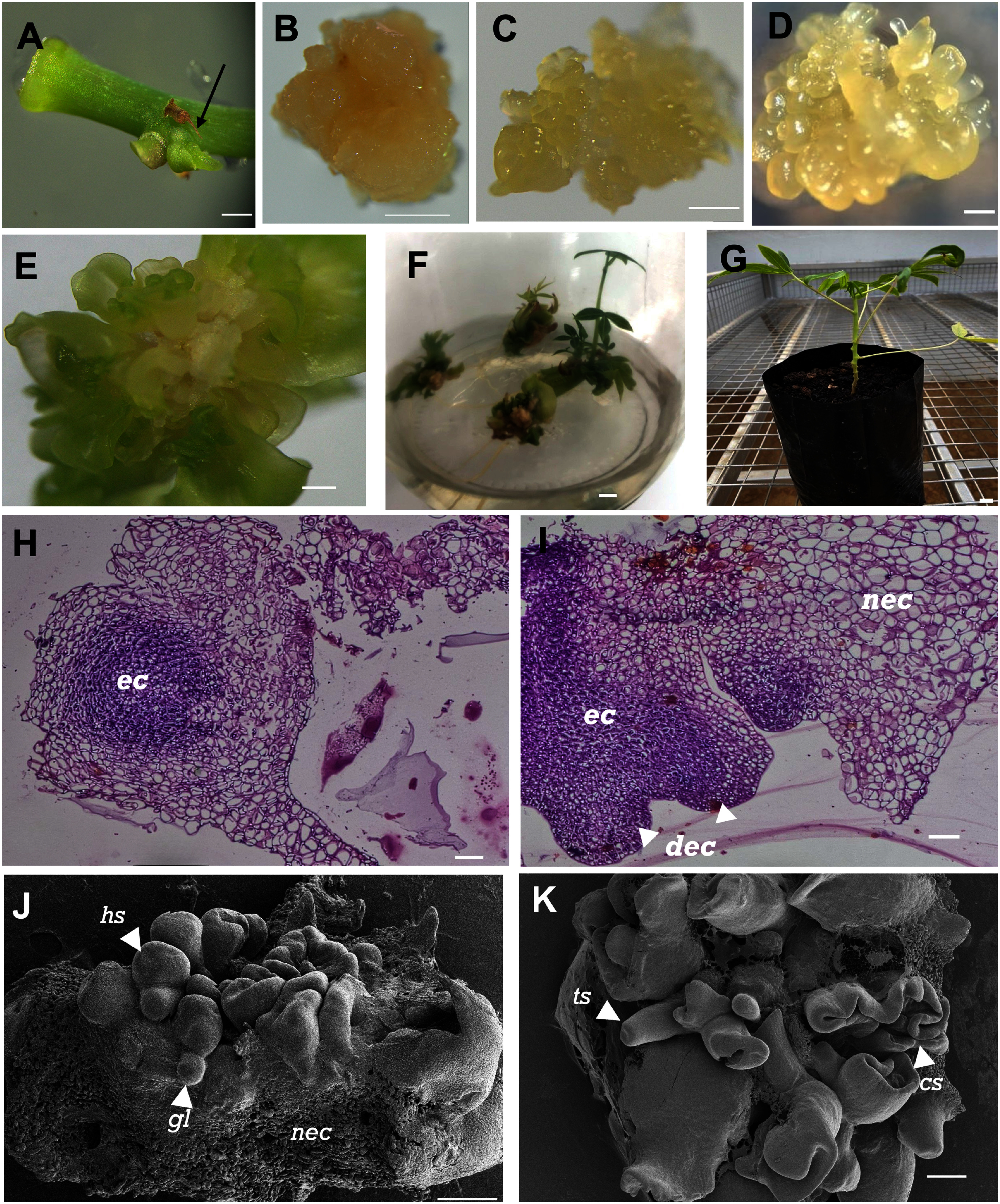
Figure 1. Morphological, histochemical, and cryo-EM images of the stages of somatic embryogenesis induction and development in Menti genotype representing the three assessed cassava genotypes. (A) Induced axillary bud (AB) explant (indicated by an arrow) 4 days after initial culturing on cassava axillary medium. (B) Somatic embryogenic calli (SEC) from an induced AB explant on a callus induction medium (CIM). (C) Early somatic embryos develop on CIM. (D) Later stages of somatic embryos cultured on CIM. (E) Cotyledonary-stage somatic embryo on Murashige and Skoog+NAA (MSN) medium. (F) Somatic embryo to shoot conversion on Murashige and Skoog medium. (G) Acclimatized cassava plantlets in polybags. Representative histology of (H) the initial embryogenic callus tissues (iec) and (I) differentiated embryogenic callus (dec) in the early somatic embryo (SE) development from somatic embryogenic callus (SEC). The non-embryogenic callus (nec) tissues are incapable of regenerating SEs. Cryogenic electron microscope images of (J) 2-week-old SEC with globular (gl) and heart-shaped (hs) somatic embryos, and (K) 4-week-old SEC differentiating into torpedo shaped (ts) and cotyledonary stage (cs) somatic embryos. Bars: A–E and H–K: 500 μm or 0.05 cm; F–G: 1 cm.

The explants placed on CIM started to show responses within 2 weeks after the initiation of culture. The induced axillary bud explants in CAM (Figure 1A) were observed to initiate SEC (Figure 1B). SEC then develop into early SEs (Figure 1C). SEC characterized by compact, irregular, and clumped shape. While early SEs more organized, characterized by globular shape, a smooth, and dense surface. On CIM, specific proportions of planted leaf lobe and leaf explants swelled at the cut edges prior to callus formation, with swelling and callus formation eventually spreading across the explant surface. Comparatively, the stem and petiole explants placed on CIM were found to be characterized by localized swelling at the cut sites and failed to develop into unorganized tissues (callus).

Unorganized tissue formation from the explants was considered indicative of SEC formation and the differentiated structure that arises from the SEC will develop into SEs. Morphologically, SEC looks like a little callus that is normally compact, creamy white or yellowish and sometimes has glossy nodular areas that show where the embryo is forming. In contrast, SEs exhibit organized developmental stages, which can be visually distinguished as globular, heart-shaped, torpedo, and cotyledonary stages. These embryos are usually smooth, have a symmetrically shaped, and show clear apical-basal polarity, distinguishing them from the unorganized structure of the callus.

Results of ANOVA indicated that genotype significantly affect the percentages of somatic embryo-forming calli and the total number of SE (Table 1). However, there were insignificant genotype effects on the percentage of callus-forming explants. Numbers in Table 1 are the averages across explant and auxin types to present the significant effects of the genotype. In all explant and auxin type combinations, the percentages of somatic embryo-forming calli and the total number of SE for Menti (Table 1) are the highest (57.5%) than Manggu and Nangka (38.6% and 38.9%, respectively). Numbers in Table 2 are the averages across genotype to present the significant effects of explant and auxin type combinations. For all genotypes, the percentage of callus-forming explants, percentage of somatic embryo-forming callus and the total number of SE of AB explant on Picloram supplemented medium are the best than the other explant and auxin type treatment combination.

**Table 1:** Table 1. The effect of a genotype on the percentages of callus-forming explant (PCFE) and somatic embryo-forming callus (PSEFC), and total number of somatic embryos (TNSEs) for the three cassava genotypes.

Genotype	PCFE (%)	PSEFC (%)	TNSEs
Menti	32.78^a^	57.5^a^	16.2^a^
Manggu	27.28^a^	38.6^b^	10.0^b^
Nangka	27.78^a^	38.9^b^	7.8^b^

Data are presented as mean (*n*=10, 3 replications) across explant and auxin types to show the main effect of genotype. Values followed by different lowercase letters within the same column indicate significant differences among genotypes based on Tukey’s honest significant difference test at α=0.05.

**Table 2:** Table 2. The effects of explant and auxin types on the average percentages of callus-forming explant (PCFE) and somatic embryo-forming callus (PSEFC), and total number of somatic embryos (TNSEs) for the three cassava genotypes.

Explant types	Genotypes	PCFE (%)		PSEFC (%)		TNSEs	
		Picloram	2,4-D	Picloram	2,4-D	Picloram	2,4-D
AB	Menti	100.0±0.0	30.0±10.0	93.3±5.7	23.3±5.7	202.3±15.5	32.7±12.8
	Manggu	100.0±0.0	33.3±5.7	93.3±11.5	16.7±5.7	135.7±15.0	15.3±6.3
	Nangka	100.0±0.0	33.3±5.7	73.3±5.7	20.0±0.0	140.0±11.2	18.3±1.1
Average AB		100.0^aA^	32.2^bA^	86.7^aA^	20.0^bA^	159.3^aA^	22.1^bA^
LL	Menti	70.0±10.0	56.7±5.7	56.7±5.7	16.7±5.7	88.0±11.3	19.0±7.9
	Manggu	56.7±5.7	26.7±5.7	43.3±5.7	10.0±0.0	69.3±11.8	8.7±0.5
	Nangka	56.7±5.7	36.7±5.7	43.3±5.7	20.0±0.0	53.3±8.7	15.3±1.5
Average LL		61.1^aB^	40.0^bA^	47.8^aB^	15.5^bA^	70.2^aB^	14.3^bA^
Le	Menti	30.0±10.0	26.7±5.7	6.7±5.7	0.0±0.0	3.0±5.2	0.0±0.0
	Manggu	20.0±0.0	13.3±5.7	3.3±5.7	0.0±0.0	2.7±4.6	0.0±0.0
	Nangka	23.3±5.7	13.3±5.7	10.0±10.0	0.0±0.0	6.3±6.5	0.0±0.0
Average Le		24.5^aC^	17.8^aB^	6.7^aC^	0.0^aB^	4.0^aC^	0.0^aB^

Data are presented as mean (*n*=10, 3 replications) ± standard deviation (SD). AB, induced axillary bud; LL, leaf lobe; Le, leaf explants. For each explant type, mean values followed by different lowercase letters indicate significant differences between Picloram and 2,4-D based on Tukey’s honest significant difference test at α=0.05. For each auxin type, mean values followed by different uppercase letters indicate significant differences among explant types (AB, LL, and Le) based on the same test.

Apart from localized swelling at the cut site, none of the cultured stem and petiole explants showed any clear response. Therefore, the respective percentage of callus-forming explants, percentage of somatic embryo-forming callus and the total number of SE are not presented in Table 2. The interaction effects between auxin and explant types were found to have a statistically significant effect on the percentage of callus-forming explants. Induced axillary bud and leaf lobe explants of three cassava genotypes show a significantly higher percentage of callus-forming explants on CIM supplemented with Picloram than 2,4-D (Table 2). On the other hand, percentage of callus-forming explants from leaf on Picloram are similar to those of 2,4-D (Table 2). For the three genotypes cultured on CIM supplemented with Picloram, the percentage of callus-forming explants from induced axillary bud explants are the highest (100%), followed by those of leaf lobe (61.1%) and leaf (24.4%). On the other hand, for the three genotypes cultured on CIM supplemented with 2,4-D, the percentage of callus-forming explants from leaf lobe (40%) and induced axillary bud (32.2%) explants are only significantly different to those of leaf explants (17.8%) (Table 2). This results indicated that Picloram is more effective than 2,4-D in inducing callus formation, particularly when using induced axillary bud and leaf lobe explant.

The percentages of somatic embryo-forming calli of induced axillary bud explants from the three genotypes had the highest values (73.3–93.3%) in a CIM medium supplemented with Picloram. In contrast, the percentages of somatic embryo-forming calli of induced axillary bud explants ranged from 16.7% to 23.3% on CIM medium supplemented with 2,4-D (Table 2). For the three genotypes, the percentages of somatic embryo-forming calli generated from leaf lobe explants on medium with Picloram (43.3–56.7%) or 2,4-D (10.0–20.0%) was lower than that of induced axillary bud explants, whereas the lowest values (3.3–10.0% for Picloram and 0.0% for 2,4-D) were recorded for the leaf explants (Table 2). These results indicate that Picloram is more effective than 2,4-D in promoting somatic embryo formation, particularly when using induced axillary bud explants.

The response of each explant type and combination of cassava genotypes to the total number of somatic embryos depended on the supplementation of Picloram or 2,4-D in the medium (Table 2). On CIM supplemented with Picloram, induced axillary bud explants of the three cassava genotypes generated the highest average of the total number of SE (159.3), while the leaf explant is the lowest (4) (Table 2). On the other hand, induced axillary bud or leaf lobe cultured on CIM supplemented with 2,4-D shows an insignificant average of the total number of SEs. Moreover, leaf of the three genotypes responded the lowest on CIM supplemented with 2,4-D (Table 2). The highest total numbers of SEs yielded from SEC from axillary bud explants cultured on CIM supplemented with Picloram are 202.3 (Menti), 135.7 (Manggu), and 140.0 (Nangka), respectively (Table 2). Meanwhile, the lowest total number of SEs are from the leaf explants cultured on CIM supplemented with Picloram or 2,4-D (Table 2). Among the three assessed genotypes, induced axillary bud explants were consistently characterized by the best performance in the total number of SEs, followed by leaf lobe and leaf, with the highest values being obtained for the Menti genotype, followed by the Nangka and Manggu genotypes (Table 2). Therefore, the Menti genotype was selected as a representative example for the somatic embryo regeneration shown in Figure 1. Notably, no apparent morphological differences in callus or somatic embryo structures were observed among the three genotypes.

### Plant regeneration from somatic embryogenic callus

As shown in Figure 1, the regeneration of cassava somatic embryogenesis callus follows the standard SE development process, which is characterized by clearly defined embryogenic stages. Later stages of somatic embryo (Figure 1D) developed into the cotyledonary-stage embryos (Figure 1E) after 1 to 2 weeks on the MSN medium. The cotyledonary-stage embryos turned green, forming multiple green shoot buds and then transferred to CEM to facilitate shoot development (Figure 1F). After 1 to 2 weeks, the shoots were transferred to MS medium to promote further development into plantlets, which were transferred to 24-cell trays for acclimatization. After 4 to 6 weeks, the plants were transferred to polybags (Figure 1G).

Table 3 presents data corresponding to the redifferentiation stage of somatic embryogenesis, highlighting the effects of explant and auxin types on cotyledonary-stage somatic embryo formation, somatic embryo to shoot conversion, and shoot height. Results of the analysis indicated that the genotype effects are statistically insignificant for percentages of cotyledonary-stage somatic embryo formation, percentages of somatic embryo to shoot conversion, and average shoot height. In the three cassava genotypes, induced axillary bud explants cultured on medium supplemented with Picloram show a significantly higher percentages of cotyledonary-stage somatic embryo formation, somatic embryo to shoot conversion, and average shoot height than 2,4-D (Table 3). The percentages of cotyledonary-stage somatic embryo formation, percentages somatic embryo to shoot conversion, and average shoot height of leaf, petiole and stem explants cultured on Picloram or 2,4-D supplemented medium were all 0.0, and the data are not presented in Table 3.

**Table 3:** Table 3. The effects of explant and auxin types on the average percentages of cotyledonary-stage somatic embryo formation (PCSEF), somatic embryo-to-shoot conversion (PSESC), and average shoot height (ASHe) for the three cassava genotypes.

Explant types	Genotypes	PCSEF (%)		PSESC (%)		ASHe (cm)	
		Picloram	2,4-D	Picloram	2,4-D	Picloram	2,4-D
AB	Menti	34.0±5.3	18.3±5.5	49.3±3.0	15.3±14.5	5.4±0.1	2.7±2.3
	Manggu	27.3±2.1	13.3±2.5	70.0±3.0	27.7±25.4	7.2±0.2	4.3±3.7
	Nangka	22.3±2.5	29.0±3.0	51.3±4.9	30.0±17.3	5.4±0.1	5.3±0.3
Average AB		27.9^aA^	20.1^bA^	56.8^aA^	24.2^bA^	6.0^aA^	4.1^bA^
LL	Menti	23.3±8.5	19.7±3.5	47.0±9.0	8.3±14.4	5.2±1.5	1.0±1.7
	Manggu	28.7±2.1	19.3±7.3	54.7±13.2	0.0±0.0	7.1±0.2	0.0±0.0
	Nangka	14.0±6.1	13.3±7.0	23.7±6.3	0.0±0.0	6.0±0.1	0.0±0.0
Average LL		21.9^aB^	17.5^aA^	41.7^aB^	2.7^bB^	6.1^aA^	0.3^bB^

Data are presented as mean (*n*=10, 3 replications) ± standard deviation (SD). AB, induced axillary bud; LL, leaf lobe. For each explant type, mean values followed by different lowercase letters indicate significant differences between Picloram and 2,4-D based on Tukey’s honest significant difference test at α=0.05. For each auxin type, mean values followed by different uppercase letters indicate significant differences between explant types (AB and LL) based on the same test.

SEs derived from the induced axillary bud explants cultures on CIM supplemented with Picloram were characterized by the highest percentage of cotyledonary-stage somatic embryo formation (27.9%) in MSN, followed by that from the leaf lobe explants (21.9%). Meanwhile, those cultured from medium supplemented with 2,4-D had lower (20.1% for axillary bud and 17.5% for leaf lobe explants, respectively) (Table 3). Among the explant types assessed, we established that the highest SE-to-shoot conversion response in CEM was obtained for SEs from induced axillary bud explants cultured on CIM supplemented with Picloram (56.8%), which was slightly higher than the 41.7% conversion obtained for the SEs derived from the leaf lobe explants cultured on the same Picloram-supplemented CIM (Table 3). In contrast, we obtained a comparatively moderate to low percentages (24.2% for axillary bud and 2.7% for leaf lobe explants) for converting SEs derived from explants cultured on CIM supplemented with 2,4-D. For average shoot height, the matured SEs originated from leaf lobe explants cultured on CEM supplemented with Picloram were found to have the tallest shoots (6.1 cm on average), followed by the matured SEs originated from induced axillary bud explants (6.0 cm on average).

### Histological and cryo-EM analyses of somatic embryo development from embryogenic calli

Histological analysis of the three cassava genotypes, as represented by Menti genotype, provided insights into the somatic embryogenic calli (SEC) developmental stages. Representative images of the Menti genotype tissues at different stages of development are shown in Figure 1H, 1I. Observations revealed dark-stained, densely packed cells (Figure 1H), indicating initial embryogenic callus. The characteristics of this callus also indicated that the constituent cells were actively dividing and proliferating. The initial embryogenic calli developed into SEC and differentiated embryonic calli (Figure 1I). Moreover, the rounded, ball-like shape and densely packed cells are also cellular characteristics of the globular stage of SE development (Figure 1I). The non-embryogenic callus contains large, lightly stained cells with small nuclei and large vacuoles (Figure 1I), indicating that this callus type comprises less actively dividing and more dedifferentiated, non-embryogenic tissues.

Cryo-EM imaging was conducted to confirm the presence of the progressive stages of somatic embryogenesis in calli derived from the Menti genotype. The images thus obtained show the morphological development of the SEs of cassava at 2 and 4 weeks after callus induction. Cryo-EM analysis indicated the presence of the morphological and cellular characteristics of globular and heart-shaped (Figure 1J), torpedo-shaped, and cotyledonary stages of SE development (Figure 1K). The images clearly show the early stages of spherical SEs and indicate the initiation of SE development on CIM (Figure 1J). The heart-shaped embryos represent a more advanced SE development stage, progressing from the globular stage (Figure 1J). A non-embryogenic callus was formed at the basal region of the explants. This tissue cannot differentiate into SEs and often appears as dedifferentiated and unorganized cells (Figure 1J). The torpedo-shaped SEs (Figure 1K), which occur at later stages in SE development, represent elongated and polarized structures characteristic of advanced differentiation. The cotyledonary-stage SEs signify the transition toward full SE maturation (Figure 1K).

## Discussion

The availability of SEC and SE regeneration methods is essential for enabling progress in modern biotechnological procedures for cassava improvement (Berhanu and Feyissa 2020; Danso et al. 2010). Effective SEC induction and SE regeneration methods facilitate cellular and molecular biotechnological approaches, such as producing disease-free (bacterial-, fungal-, and viral-free) planting materials (Nkaa et al. 2013). Subsequently, disease-free cassava can be massively vegetatively propagated based on the developed SEC induction and SE regeneration methods (Chavarriaga-Aguirre et al. 2016). For example, the findings of previous studies have indicated that disease-free *Vitis vinifera* (Malenica et al. 2020), *Piper nigrum* (Sasi and Bhat 2018), and *Citrus* spp. (Meziane et al. 2017) can be regenerated using SE, and SE-derived seedlings have been used to supply large-scale planting materials for *Musa* spp. (Natarajan et al. 2020), *Coffea arabica* (Etienne et al. 2013) and *Allium cepa* (Wu et al. 2015).

Regeneration of desirable cassava somaclonal variants should also be possible using in vitro cultures, chemical mutagenesis, and irradiation in combination with cassava SEC induction methods. Indeed, viral-resistant and stress-tolerant variants of cassava have been identified using SEC in combination with in vitro selection (Nkaa et al. 2013). The developed SEC induction method can also contribute to modern biotechnological techniques for the development of FEC cassava, such as plant genetic engineering and CRISPR/Cas9 genome editing, the application of which have been demonstrated using FEC explants (Odipio et al. 2017; Wang et al. 2022). These findings highlight the need to develop efficient methods for cassava SEC induction and SE regeneration, particularly in the case of genotypes for which such techniques have not been previously described, given that SEC induction in cassava is generally genotype-specific.

Factors influencing the success of cassava SE induction include basal medium composition, supplementation of media with auxins for SEC induction and subsequent plantlet regeneration, the explant types utilized for callus initiation, and cassava genotypes (Ajijah et al. 2016; Ibrahim et al. 2013; Rossin and Rey 2011). Moreover, morphological and developmental stages of explants can also influence the efficacy of SE initiation (Schädel et al. 2010; Syombua et al. 2019).

In this study, we established that the explant type influences the success of cassava SE induction and subsequent plantlet development. Among the five assessed cassava explants, axillary buds obtained from in vitro-cultured cassava were characterized by the highest capacity for SE formation. The cassava meristematic tissues in axillary bud explants were more responsive to SE regeneration on CIM supplemented with Picloram or 2,4-D than in the other explant types. These findings are consistent with those previously reported, indicating that meristematic tissues are the most effective explant sources for initiating somatic embryogenesis (Bull et al. 2009; Rossin and Rey 2011; Zainuddin et al. 2012).

Although not as effective as induced axillary buds, leaf lobes and leaves can be used as alternative explants for inducing somatic embryogenesis in cassava (Nyaboga et al. 2015; Taylor et al. 2012). In three cassava genotypes grown locally in Indonesia, induced axillary buds are more effective in inducing somatic embryogenesis than leaf lobes or leaves. SE induction has also been reported in cassava accessions from South America, Asia, and Africa using different explant types (Atehnkeng et al. 2006; Feitosa et al. 2007; Saelim et al. 2006). These findings indicate the importance of selecting appropriate meristematic tissues for the efficient induction of somatic embryogenesis. We also established that auxin-supplemented media are essential for initiating and subsequently regenerating cassava SEs. We found cassava leaf explants developed unorganized calli and SEs on auxin-supplemented CIM. However, these SEs failed to regenerate into plantlets. When cultured on an auxin-supplemented medium, cassava stem and petiole explants were observed to undergo swelling. However, they did not develop into unorganized calli. Similar responses to auxin-containing media have been reported for SE induction in *Quercus robur* (Cuenca et al. 1999) and *Solanum nigrum* (Xu et al. 2014).

Callus formation initially occurs at the edges of wounded cassava leaves in response to wounding-induced cell dedifferentiation. The swollen tissues eventually become an unorganized callus covering the entire leaf surface. Contrastingly, hard and woody tissues, such as stem explants, are typically less responsive to callus induction, with the responses of wounded cassava stems and petioles being limited to localized swelling. Similar responses have been observed in wounded petiole explants of *Olea europaea* without callus development (Narváez et al. 2019). Consequently, cassava stems and petioles are not recommended as explants for cassava SEC.

Furthermore, the fact that leaves are less lignified than stem and petiole explants tends to make these more responsive to auxins, which can be readily programmed to undergo dedifferentiation (Fletcher et al. 2011; Schädel et al. 2010). Such conditions may induce differential responses among leaf, stem, and petiole explants (San-José et al. 2010; Schädel et al. 2010). Moreover, injuring cassava stems and petiole segments results in the extrusion of latex, which can hamper SE development. Our findings thus indicate that axillary buds are the optimal initial explants for the in vitro induction of cassava SEs. However, leaf lobes can also be an alternative if axillary buds are unavailable.

The differential responses among explant types may be associated with the levels of endogenous auxins, as the findings of previous studies have indicated that different explants can differ in their response to the same medium supplemented with the auxins. In this regard, it has been established that auxins are essential for promoting somatic embryonic development (Lincy et al. 2009) and can induce the reorganization of somatic cells with a predetermined fate to undergo dedifferentiation and express embryogenic competence (Danso et al. 2010).

Picloram is a synthetic auxin known for its efficacy in inducing somatic embryogenesis in various plant species, including date palm (Mervat et al. 2024) and *Lycium barbarum* L. (Khatri and Joshee 2024). Picloram has been established as a functionally effective auxin in cassava. We demonstrated in this study that when used as a CIM supplement for SE induction, Picloram outperformed 2,4-D in three Indonesian cassava genotypes. Such results are consistent with previously reported beneficial effects of Picloram (Berhanu and Feyissa 2020; Danso et al. 2010).

Histological and cryo-EM observations confirmed the presence of different developmental stages of SE, including the globular, heart-shaped, torpedo-shaped, and cotyledonary stages. Understanding the cellular characteristics of the different developmental stages of SEs and the difference between embryogenic and non-embryogenic calli will enable researchers to develop more efficient methods for SE formation and development. Having thus developed effective methods for cassava SEC induction and subsequent plantlet regeneration, cellular and molecular biotechnological procedures for improving cassava cultivars, such as providing large numbers of disease-free planting materials, generating desirable somaclonal variants or mutants, genetic engineering, and CRISPR/Cas9 genome editing, are possible, which should accordingly be the focus of future research on cassava production in Indonesia.

In summary, methods for effective SEC induction from three locally grown cassava genotypes in Indonesia were developed using induced axillary bud explants cultured on CIM supplemented with 12 mg l^−1^ Picloram. SEC proliferation was performed by subculturing these calli on fresh Picloram-supplemented CIM. Subsequently, the cassava SEC developed into mature somatic embryos (SEs). Following SE germination, it can be used to regenerate entire cassava plantlets. The SEC induction and subsequent SE and plantlet regeneration methods developed in this study open the possibility of conducting cellular and molecular biotechnological procedures for improving cassava production.

## Abbreviations

2,4-D: 2,4-dichlorophenoxyacetic acid
CAM: cassava axillary medium
CBM: cassava basic medium
CEM: cassava elongation medium
CIM: callus induction medium
MS: Murashige and Skoog
MSN: Murashige and Skoog+NAA for somatic embryo emergence
NAA: naphthalene acetic acid
OES: organized embryogenic structure
SE: somatic embryo
